# Live-cell imaging of endocytosed synaptophysin around individual hippocampal presynaptic active zones

**DOI:** 10.3389/fncel.2023.1277729

**Published:** 2023-10-19

**Authors:** Hiromitsu Tanaka, Junichiro Funahashi, Tomoo Hirano

**Affiliations:** ^1^Department of Biophysics, Graduate School of Science, Kyoto University, Kyoto, Japan; ^2^Department of Developmental Biology, Graduate School of Medicine, Chiba University, Chiba, Japan

**Keywords:** synaptic vesicle, endocytosis, clathrin, TIRFM, synaptophysin (SYN), cytomatrix at the active zone-associated structural protein (CAST), active zone (AZ)

## Abstract

In presynaptic terminals 4 types of endocytosis, kiss-and-run, clathrin-mediated, bulk and ultrafast endocytosis have been reported to maintain repetitive exocytosis of neurotransmitter. However, detailed characteristics and relative contribution of each type of endocytosis still need to be determined. Our previous live-cell imaging study demonstrated individual exocytosis events of synaptic vesicle within an active-zone-like membrane (AZLM) formed on glass using synaptophysin tagged with a pH-sensitive fluorescent protein. On the other hand, individual endocytosis events of postsynaptic receptors were recorded with a rapid extracellular pH exchange method. Combining these methods, here we live-cell imaged endocytosed synaptophysin with total internal reflection fluorescence microscopy in rat hippocampal culture preparations. Clathrin-dependent and -independent endocytosis, which was seemingly bulk endocytosis, occurred within several seconds after electrical stimulation at multiple locations around AZLM at room temperature, with the locations varying trial to trial. The contribution of clathrin-independent endocytosis was more prominent when the number of stimulation pulses was large. The skewness of synaptophysin distribution in intracellular vesicles became smaller after addition of a clathrin inhibitor, which suggests that clathrin-dependent endocytosis concentrates synaptophysin. Ultrafast endocytosis was evident immediately after stimulation only at near physiological temperature and was the predominant endocytosis when the number of stimulation pulses was small.

## Introduction

In presynaptic terminals, the rapid and repetitive exocytosis of synaptic vesicles occurs. Because the number of synaptic vesicles is limited, efficient synaptic vesicle retrieval processes including endocytosis are necessary. Morphological observations using electron microscopy (EM), electrophysiological capacitance measurements of the cell-surface area and live-cell imaging have been performed (Jin et al., 2019), and four types of endocytosis have been reported. After the full-fusion type of exocytosis, clathrin-mediated endocytosis (CME) and clathrin-independent one occurs in the vicinity of an active zone (Granseth et al., 2006; Chanaday et al., 2019). The latter include activity-dependent bulk endocytosis (ADBE) and ultrafast-type endocytosis (UFE). In ADBE a large endosome is produced after repetitive activation of a presynaptic neuron, and UFE occurs immediately after the presynaptic exocytosis (Cheung and Cousin, 2013; Watanabe et al., 2013; Soykan et al., 2017). Kiss-and-run endocytosis (K&R), in which the vesicle and cell membrane fuse transiently has also been reported (Zhang et al., 2009; Ge et al., 2022), although its existence in neuronal presynaptic terminals is controversial (Granseth et al., 2006; Wu et al., 2014).

Each of the above methods to study presynaptic endocytosis has merits and demerits. EM captures images with ultra-high resolution but at only one-time point for each image (Watanabe et al., 2013, 2014; Jin et al., 2019). Recording the membrane capacitance measures the change of the surface area of the presynaptic terminal with high temporal resolution (Delvendahl et al., 2016; Jin et al., 2019), but it does not provide information about the location or morphological properties of each endocytosis type. Live-cell fluorescence imaging records the movement and location of released synaptic vesicle proteins even at a single synapse, and imaging trials can be repeated in the same cell, but this approach has limited spatial resolution compared with EM and low time resolution compared with electrophysiological capacitance measurements. Overall, the distribution of each endocytosis type around individual active zones has not been demonstrated at multiple time points in living neurons.

In the present study, we used total internal reflection fluorescence microscopy (TIRFM), which allows the observation of fluorescent molecules with a high signal-to-noise ratio by limiting the depth of the excitation field to approximately 100–200 nm (Axelrod, 2001; Toomre and Manstein, 2001), to improve the quality of the fluorescent images of endocytosed presynaptic proteins. In our previous studies, to efficiently visualize fluorescent molecules at active zones with TIRFM, we induced the formation of active zone-like membranes (AZLM) parallel to the glass surface by coating with neuroligin (NLG). This synaptic adhesion molecule induces presynaptic differentiation through binding with presynaptic neurexin (NRX) (Funahashi et al., 2018). With this setup, we recorded single exocytosis events using a synaptic vesicle protein, synaptophysin (Syp), tagged with a pH-sensitive variant of green fluorescent protein (GFP) called super-ecliptic pHluorin (SEP). SEP is non-fluorescent in the low pH intra-vesicular solution but becomes fluorescent after exposure to the neutral pH of the extracellular solution (Miesenböck et al., 1998; Kavalali and Jorgensen, 2013). We also visualized endocytosis events of glutamate receptors labeled with SEP in the postsynaptic membrane (Fujii et al., 2017, 2018). In those studies, the extracellular pH was changed intermittently and locally to an acidic condition using a U-tube system. At pH 6.0, cell-surface SEP signals were quenched, and only signals could be detected from recently endocytosed vesicles in which acidification had not been completed. pH-sensitive fluorophores and extracellular pH exchange have been used to study endocytic processes (Merrifield et al., 2005; Rosendale et al., 2017). Combining TIRFM, AZLM formation and rapid pH exchange using U-tube, in the present study, we observed endocytosed Syp-SEP signals after an electrical field stimulation to trigger the exocytosis of synaptic vesicles and analyzed the distribution patterns of the endocytosed Syp-SEP signals around AZLM at several time points. We show the distributions of Syp-SEP during and after exo- and endocytosis around AZLM and analyze the characteristics of each type of endocytosis.

## Materials and methods

### Animals

All experimental procedures were carried out in accordance with the National Institute of Health guide for the care and use of laboratory animals and the ethical guidelines on animal experimentation of Kyoto University, and were approved by the local committee for handling experimental animals in the Graduate School of Science, Kyoto University.

### Primary cell culture and transfection

**Figure 1:**
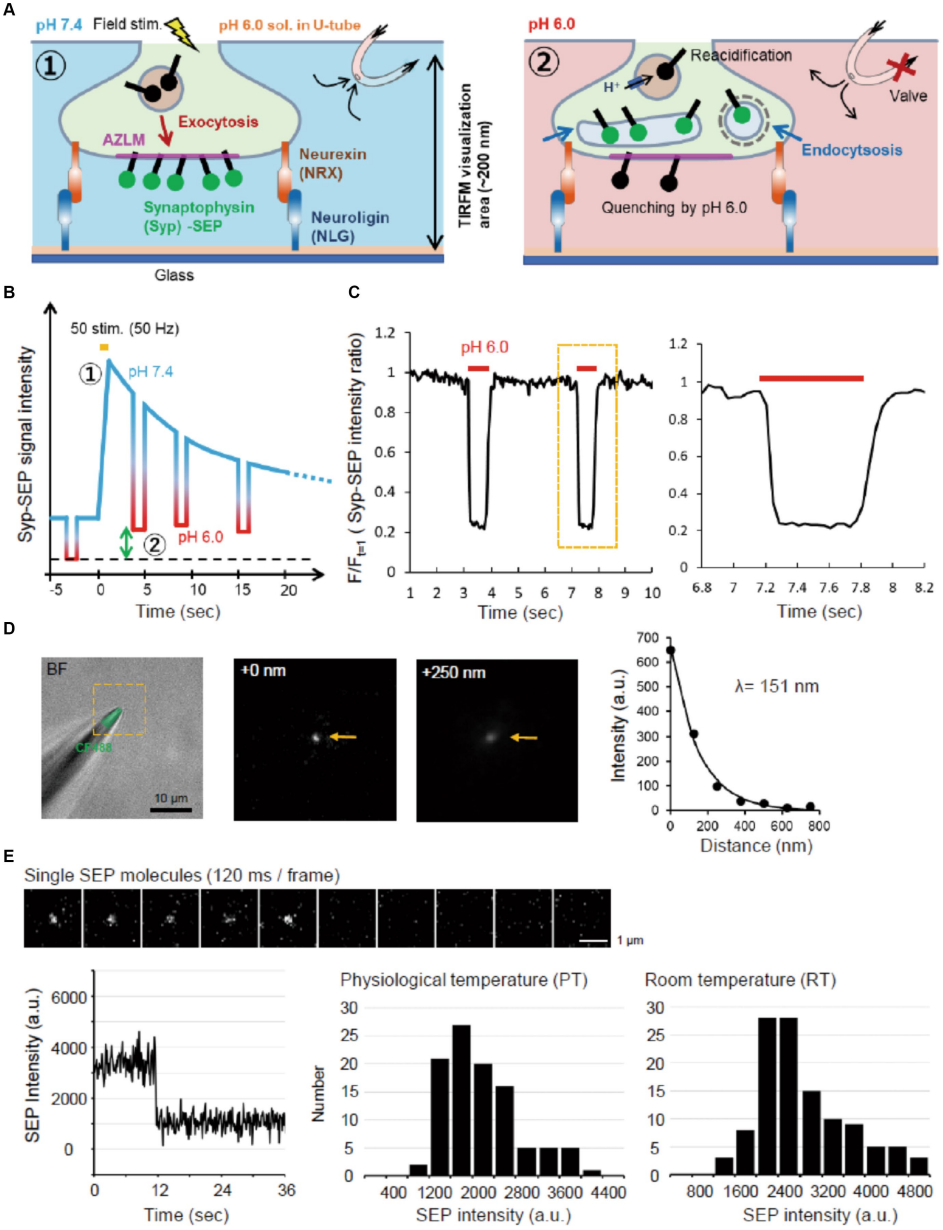
Experimental scheme and system. **(A)** Schematic diagram of AZLM and pH exchange method. Arrows around top right U-tube indicate directions of solution flow. **(B)** Experimental design. An electrical field stimulation (50 pulses, 50 Hz) was applied to induce the exocytosis of synaptic vesicles containing Syp-SEP [① in **(A,B)**]. The endocytosed Syp-SEP was observed at each time point by repeating a pH exchange from 7.4 to 6.0 [② in **(A,B)**]. The green two-way arrow corresponds to the amount of endocytosed Syp-SEP. **(C)** The pH-exchanging speed. The right graph shows an enlargement of the region enclosed by the dotted yellow line. **(D)** Observation area of TIRFM in the Z-axis. The tip of a glass electrode containing CF488 dye (left) was observed under TIRFM. The middle and right TIRFM images are enlargements of the yellow-dotted square region in the left bright field + fluorescence image at 0 and 250 nm from the glass surface. Arrows indicate the electrode tip. The length constant of the visualization depth of TIRFM was calculated to be 151 nm (right). **(E)** Single molecule analysis of SEP. Sequential images of the SEP signal recorded every 120 ms with TIRFM (top). The time course of the SEP fluorescence intensity, showing single-step photobleaching characteristic of a single molecule (bottom left). Distribution of single SEP fluorescence intensities at PT (bottom middle) and RT (bottom right).

The methods for preparing the primary culture of hippocampal neurons and transfection of cDNA were described previously (Tanaka and Hirano, 2012; Tanaka et al., 2014). Briefly, hippocampi were dissected out from E18-P0 Wistar rat embryos, treated with 0.25% trypsin (Thermo Scientific, 15090-046) and dissociated by trituration with a fire-polished Pasteur pipette. Dissociated cells were seeded on poly-D-lysine-(Merck, P7280 and P6407) and NLG-coated glass (Figure 1A) in Neurobasal medium (Thermo Scientific, 21103-049) containing 1% penicillin–streptomycin (Thermo Scientific, 15140-122), 500 μM glutamine (Merck, G6392) and 2% B27 Supplement (Thermo Scientific, 17504-044).

### DNA constructs

Expression vectors for rat synaptophysin labeled with SEP (Syp-SEP), cytomatrix at the active zone-associated protein (CAST) labeled with TagRFPt (CAST-RFP), neuroligin1 with splice insertion A labeled with human immunoglobulin-Fc region (NLG-Fc) and neurexin1β without splice insertion 4 labeled with HA tag (NRX) were prepared as described previously (Funahashi et al., 2018).

### Glass coating and AZLM formation

NLG-Fc was prepared from transfected HEK293 cells using nProtein A Sepharose (GE Healthcare, 17-5280-01). A piece of cover glass was first incubated with 43 μM biotinylated bovine serum albumin (Thermo Scientific, 29130) in buffer A (100 mM KCl, 5 mM MgCl_2_, 25 mM HEPES-KOH, pH7.4) at 4°C overnight (Tanaka et al., 2014). Then, the glass was incubated in buffer A containing 17 mM streptavidin (Wako, 191-12851) for 1 h, followed by incubation in buffer A containing biotin-conjugated anti-human IgG (1:100; Jackson ImmunoResearch, 109-065-098) for 1 h at RT. Then, the glass was washed and further incubated in buffer A containing 3–5 mg/mL NLG-Fc for 5 h. Expression vectors for Syp-SEP, CAST-RFPt and NRX were transfected into neurons after 10–15 days *in vitro* (DIV) with Lipofectamine 2000 (Thermo Scientific, 11668-019) (Funahashi et al., 2018). Half of the medium was replaced with fresh prewarmed medium 4–6 h after the transfection. AZLM was formed, and all imaging experiments were carried out 2–3 days after the transfection.

### Live-cell imaging and electrical field stimulation

The TIRFM imaging system was composed of an inverted fluorescence microscope (Olympus, IX71) equipped with a 100× (NA 1.49) or 150× (NA 1.45) TIRFM objective lens and 1.6× intermediate lens, EM-CCD camera (Andor, iXonEM+ DU-897), 488 nm laser (Melles Griot, 85-BCD-020) and 561 nm laser (Coherent, Sapphire 561LP). Changes in Syp-SEP signals before and after the electrical stimulation were recorded with the camera using a 40 ms exposure time and the 100× objective lens. When we wanted higher resolution images, Syp-SEP and CAST-RFP images were acquired for 120 ms with the 150× objective lens and 1.6× intermediate lens using a dual-color filter (Semrock, FF01-523/610-25). Imaging was performed in extracellular solution (120 mM NaCl, 4 mM CaCl_2_, 1 mM MgCl_2_, 10 mM glucose, 10 mM HEPES-KOH, pH 7.3) at RT or PT. The temperature of the experimental room was controlled by air conditioning. The pH was changed intermittently, and the duration of the pH 6.0 condition was 700 ms. Within this pH 6.0 period we recorded 5 high resolution images and the later 4 were used to generate an averaged image. An electrical field stimulation (duration 1 ms, 20–26 V/cm, 5, 20, 50 or 125 pulses at 50 Hz) was applied to the cultured neurons between platinum electrodes (Tanaka et al., 2014). To observe clathrin-independent endocytosis, pitstop 2 (pit2) was added at a final concentration of 10 μM, and imaging was performed 10 min later. Pit2 was used here to inhibit clathrin-dependent processes, because it was possible to record Syp-SEP images in a same preparation before and after its application. Previous studies also used knockdown of clathrin by RNAi or overexpression of a dominant negative form of clathrin-related protein (Granseth et al., 2006), but these manipulations take much longer time and recording endocytosis before and after the manipulation in a same cell is very difficult if not impossible.

To measure the observation depth of TIRFM, the tip of the glass electrode containing CF488 (Nacalai testque, 20 μM) was raised in the Z-axis direction at 125 nm steps from the glass surface using a micro manipulator (HEKA, MP-285). The fluorescence signal decreased as the glass tip was raised with a length constant of 151 nm (Figure 1D).

The same laser intensity and illumination angle were used to record the fluorescence of single SEP molecules. SEP molecules were prepared from SEP-transfected HEK293 cells by supersonic treatment and diluted with PBS. A single SEP molecule on the glass was identified by single-step photobleaching (Figure 1E).

### U-tube system

The U-tube system was prepared as described previously (Bretschneider and Markwardt, 1999; Fujii et al., 2017). To improve the speed of the external fluid exchange (Figure 1C), the inner diameter of the U-tube hole was made smaller (6–8 μm), and the hole was placed 25–50 μm above the glass surface. Using the U-tube system, the pH of extracellular solution was changed from 7.4 to 6.0 in about 100 ms and from 6.0 to 7.4 in about 200 ms (Figure 1C). The intra-U-tube solution was the same as the extracellular solution, except that the pH was adjusted to 6.0 with 2-(N-morpholino) ethanesulfonic acid and KOH.

### Image analysis

Acquired images were analyzed using MetaMorph (Molecular Devices), R (The R Project) and Excel (Microsoft). AZLM area was determined as follows. First, the CAST-RFP image was compensated for mechanical drifts of the stage. Then, the mean plus 3 times SD of the background signal intensity in each image was set as the threshold. The CAST-labeled area ranging from 0.098 to 0.38 μm^2^ was defined as AZLM. The active zone area in the hippocampus estimated by previous EM studies is <0.18 μm^2^ (mean 0.04 μm^2^) (Schikorski and Stevens, 1997; Holderith et al., 2012). However, we recorded a fluorescence signal from a single SEP molecule in several pixels due to diffraction and scattering (Figure 1E). Therefore, we set the AZLM area slightly larger. Analysis was performed on AZLMs which showed significant Syp-SEP signal increase by 5 or 50 pulses stimulation.

The Syp-SEP area reflecting exocytosis or endocytosis was determined as follows. After drift compensation (Tanaka et al., 2014), the fluorescence values of Syp-SEP were compensated for bleaching. The bleaching rate was computed by fitting the bleach curve with a double exponential function obtained from experiments without stimulation. Then, the mean plus 3 times SD of the background signal in each image was set as the threshold. Areas (> 0.098 μm^2^) of contiguous pixels above the threshold were defined as the Syp-SEP positive area. The centroid of the fluorescence intensity of the Syp-SEP-positive area was regarded as the center of Syp-SEP-containing vesicles.

For a detailed analysis of the signal intensity, area and position of endocytosed Syp-SEP, the average of 4 consecutive high-magnification images taken from the time when the extracellular pH was changed to 6.0 was used. For example, when the pH exchange to 6.0 started 3 s after the stimulation, Syp-SEP images taken at 3.24, 3.36, 3.48 and 3.60 s were averaged and used for the quantitative analyses at 3.24 s. To exclude the Syp-SEP signal from that of organelles such as the endoplasmic reticulum, each image was subtracted from the image recorded at pH 6.0 at 3.88 s before the stimulation. If an increase in the signal was observed from the background level while the extracellular pH was 6.0, it was assumed that Syp migrated from outside the TIRFM observation area within the presynaptic terminal, and the corresponding data was excluded from the analysis. When multiple Syp-SEP containing vesicles were in close proximity, it was impossible to estimate each center. However, we tried to separate a contiguous Syp-SEP positive area containing multiple intensity peaks using watershed lines (Broadhead et al., 2016; Imig et al., 2020). Watershed lines were drawn to connect local minima that existed between peaks using MetaMorph.

For the analysis of exocytosed Syp-SEP signal intensity, images recorded at 120 ms after the end of the stimulation at pH 7.4 were used. The position of exocytosis indicates the center of the entire Syp-SEP positive area, where multiple vesicles were fused to the plasma membrane, 120 ms after the onset of the stimulation.

### Experimental design and statistical analysis

The sample size in each experiment was determined based on previous publications dealing with live-cell imaging (Fujii et al., 2018; Sposini et al., 2020). In each experiment, *N* indicates the number of Syp-SEP or CAST-RFP signal clusters and/or cells except in the single molecule fluorescence analysis. When we compared means of two groups, Mann–Whitney U test or Wilcoxon signed-rank test was used in a case the normality of data distribution and/or equality of deviations were denied by Shapiro–Wilk test and/or F-test, otherwise we performed Student’s paired t-test. In multiple comparison of means, we used Steel’s test or Steel-Dwass’s test. All values are presented as the mean ± standard error of the mean. Statistical tests were performed using Excel and Kyplot (KyensLab, ver. 5.0.3).

## Results

### Visualization of Syp-SEP around AZLM after presynaptic activation

To visualize Syp-SEP translocated to the plasma membrane upon the arrival of action potentials and also endocytosed Syp-SEP in presynaptic terminals, we applied TIRFM (Figure 1A). We cultured rat hippocampal neurons on NLG-coated glass and induced AZLM formation through NLG binding to presynaptic NRX at DIV 12–18 (Funahashi et al., 2018). Neurons were transfected with CAST labeled with tagRFPt (CAST-RFP), Syp-SEP and NRX. CAST-RFP was used as an AZLM marker (Ohtsuka et al., 2002). Then, the U-tube system was applied to this experimental system (Fujii et al., 2017) to transiently and locally change the extracellular pH from 7.4 to 6.0 around AZLM, thus quenching SEP signals on the cell surface, and internalized Syp-SEP signals by endocytosis were detected (Figures 1A–C). The extracellular pH was changed from 7.4 to 6.0 in about 100 ms and from 6.0 to 7.4 in about 200 ms (Figure 1C). The length constant of the visualization depth of the TIRFM was 151 nm (Figure 1D). Therefore, the internalized Syp-SEP signals should have come from a region about 0–200 nm above the glass surface. We also evaluated the sensitivity of the recording system by recording fluorescence from a single SEP molecule on the glass surface (Figure 1E). A single SEP molecule was identified by one-step bleaching, and the fluorescence intensity was 2,900 ± 77 (*n* = 114 points) at room temperature (RT, 21–23°C) and 2,200 ± 71 (*n* = 102 points) at near physiological temperature (PT, 31–32°C) on an arbitrary scale of our experimental system.

**Figure 2:**
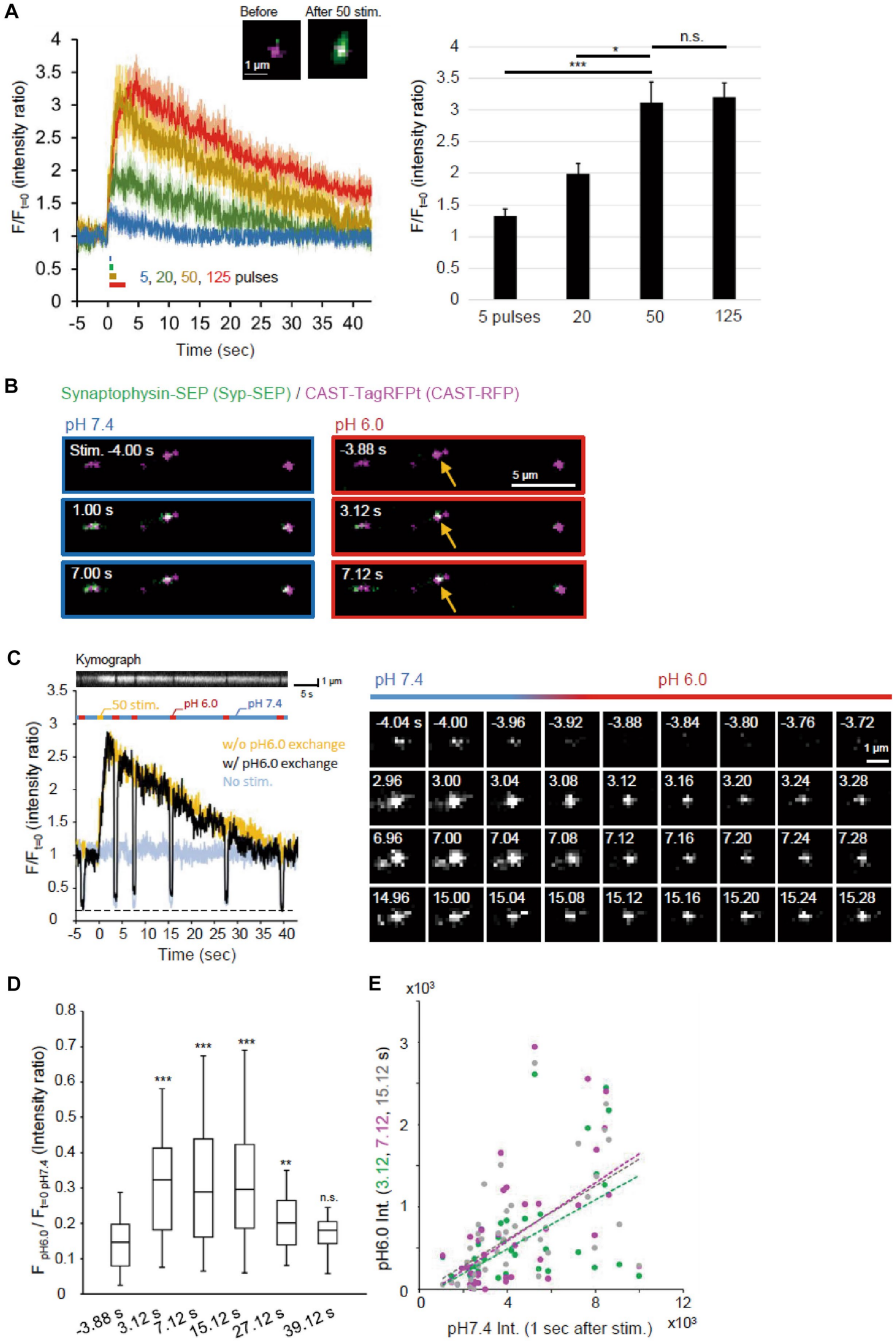
Visualization of endocytosed Syp-SEP. **(A)** Release of Syp-SEP induced by electrical field stimulation. In the left, averaged traces of Syp-SEP fluorescence intensity (means ± standard errors) before and after 5 (11 cells), 20 (12 cells), 50 (16 cells) or 125 (23 cells) pulses stimulation are presented with representative images of CAST-RFP (magenta) and Syp-SEP (green) before and after 50 pulses stimulation. In the right, the peak Syp-SEP signal intensities after 5, 20, 50 or 125 pulses stimulation normalized by the intensities before the stimulation are presented. The amount of exocytosed Syp-SEP increased almost proportionally to the number of stimulation pulses (up to 50 pulses). **(B)** Representative images of Syp-SEP and CAST-RFP before and after the 50 pulses stimulation at pH 7.4 and 6.0. Arrows indicate Syp-SEP signal at pH 6.0 observed only after the stimulation. **(C)** An example of the signal intensity of Syp-SEP before and after the 50 pulses stimulation with (black) or without (yellow) pH 6.0 exchange (left graph). Kymograph showing Syp-SEP with pH exchange is presented on the top. We set the timing of pH change to 6.0 so that whole time course of endocytosis occurrence could be clarified and that later pH change to 6.0 would show the Syp-SEP endocytosed after the previous pH exchange, although we know that the Syp-SEP signal at pH 6.0 detected at 7 s include some signal endocytosed before 3 s as described in results. The light blue trace shows the Syp-SEP signal intensity without any stimulation. Right images show Syp-SEP fluorescence before (−) and after (+) the stimulation at pH 7.4 or 6.0. **(D)** Quantification of the Syp-SEP signal intensities at pH 6.0 at various times normalized by the intensity at pH 7.4 before the stimulation (3.12 s, *n* = 43 clusters, 10 cells, 7.12 s, *n* = 43 clusters, 10 cells, 15.12 s, 40 clusters, 10 cells, 27.12 s, 39 clusters, 9 cells, 39.12 s, *n* = 41 clusters, 10 cells). **(E)** Correlation of Syp-SEP signal intensities at pH 7.4 at 1 s after the stimulation and at pH 6.0 at 3.12, 7.12 or 15.12 s after the stimulation (3.12 s, *r* = 0.54, 7.12 s, *r* = 0.56, 15.12 s, *r* = 0.58), *, **, and *** indicates *p* < 0.05, 0.01, and 0.001 respectively, n.s., not significant.

An electric field stimulation (50 Hz, 50 pulses) was applied to cultured neurons so that a large number of synaptic vesicles were fused to AZLM at RT, and an apparent increase in the Syp-SEP signal was detected (Figure 2A) (Funahashi et al., 2018). When the number of stimulation pulses was reduced from 50 to 20 or 5, the increase in the Syp-SEP signal decreased in proportion to the number of stimulation pulses (*p* < 0.05 and 0.001, Steel’s test). Conversely, the Syp-SEP signal intensity did not increase proportionally when the number of pulses was increased to 125 (Figure 2A), suggesting that the amount of release was nearly saturated at 125 and that most of the synaptic vesicles in a readily releasable pool underwent exocytosis at 50 pulses, an observation consistent with a previous report (Sakamoto et al., 2018).

Endocytosis was likely to occur during the decay of the Syp-SEP signal after exocytosis. To precisely visualize the endocytosed Syp-SEP around AZLM, the pH of the extracellular solution was changed to 6.0 repeatedly so that the fluorescence from cell-surface Syp-SEP was quenched and that only the internalized Syp-SEP signals were recorded (Figures 1A,B, 2B,C). The fluorescence signal observed at pH 6.0 was more intense at 3.12, 7.12, 15.12 and 27.12 s after the stimulation onset than before the stimulation (Figures 2C,D, *p* < 0.001 and 0.01, Steel’s test), indicating that endocytosis primarily took place at these times after exocytosis. About 40 s after the stimulation, when the Syp-SEP fluorescence signal at pH 7.4 returned to the pre-stimulation level, the signal observed at pH 6.0 almost disappeared. The Syp-SEP signal at pH 7.4 increased and decayed similarly, irrespective of the pH exchange (Figure 2C), suggesting that the pH exchange did not significantly affect the release and uptake of vesicles containing Syp-SEP. The SEP signal intensity at pH 6.0 relative to that at pH 7.4 at 1 s after the stimulation showed a near-linear relationship (Figure 2E). The intensity of endocytosed Syp-SEP was positively correlated with the intensity of exocytosed Syp-SEP. These results confirmed that the more Syp-SEP is exocytosed, the more it is endocytosed. There was weak Syp-SEP signal at pH 7.4 before the stimulation (Figure 2C, −4.04 and −4.00 s). Thus, a small portion of the Syp-SEP signal might have been derived from Syp-SEP present on the plasma membrane before the stimulation.

### Location of endocytosed Syp-SEP signals

**Figure 3:**
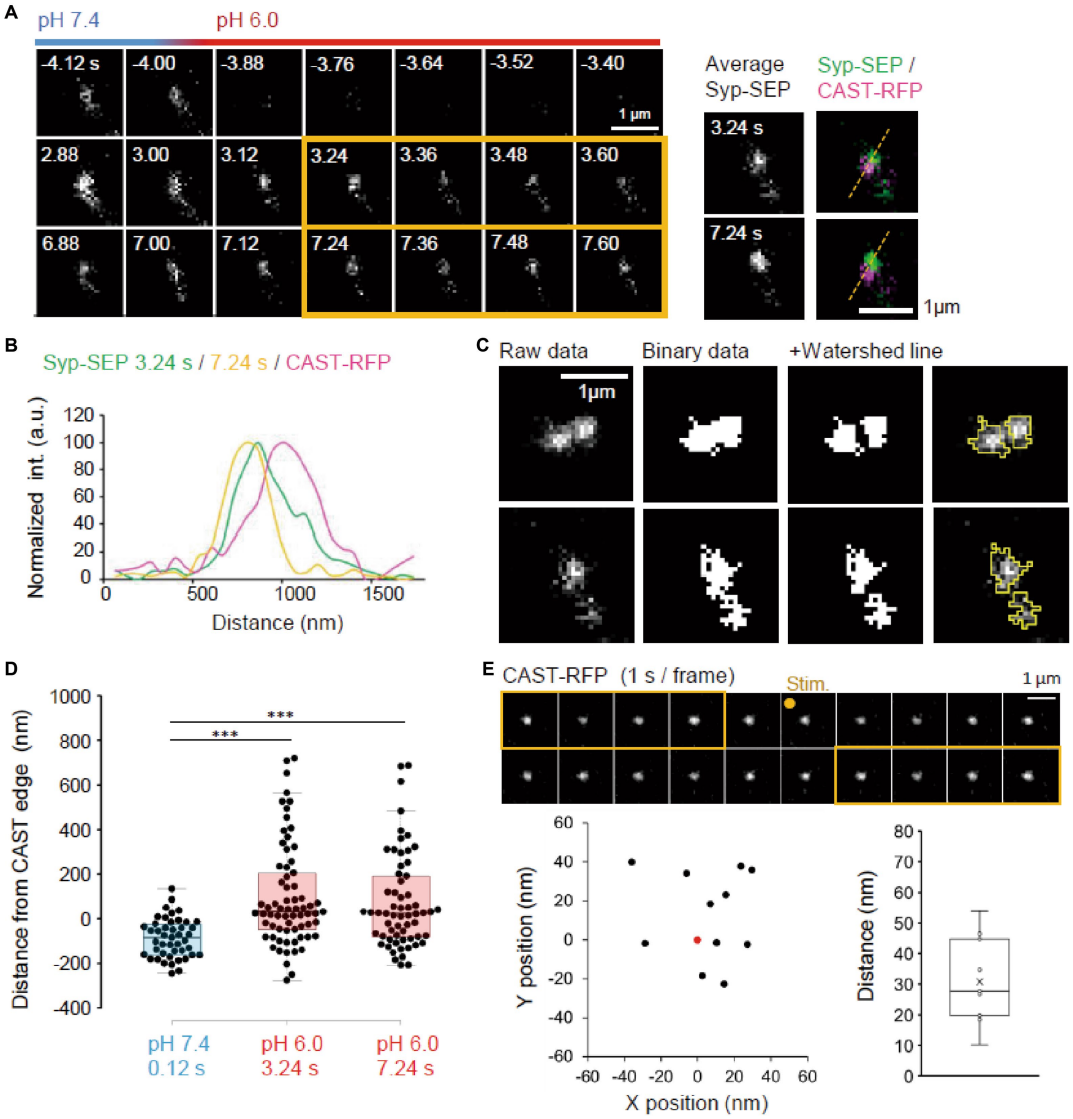
Location of endocytosed Syp-SEP signal. **(A)** Example of endocytosed Syp-SEP visualized at high magnification using 150× objective lens. The right images are averages of the images enclosed by the yellow lines and were merged with an image of CAST-RFP signals. **(B)** Line-scan of Syp-SEP or CAST-RFP signal intensities on the yellow broken lines shown in **(A)**. **(C)** Examples of binarized Syp-SEP signals and those separated by watershed segmentation. Right most images show raw data with yellow lines indicating Syp-SEP positive areas after watershed segmentation. **(D)** Location of centroids of the Syp-SEP signal positive areas at pH 7.4 at 0.12 s after the stimulation and at pH 6.0 at 3.24 or 7.24 s after the stimulation in relation to the edge of CAST-RFP (0.12 s, *n* = 45 clusters, 3.24 s, 69 clusters, 7.24 s, 62 clusters, 13 cells). **(E)** Stability of CAST-RFP signal. Representative images of CAST-RFP before and after the stimulation (top). Distribution of averaged CAST-RFP centroids at 10–14 s after the stimulation (black points) compared with that at 1–5 s before the stimulation (0.0, red point) (bottom left) (*n* = 11 clusters, 4 cells). Displacement of CAST-RFP centroids after the stimulation (bottom right). *** indicates *p* < 0.001.

Next, we imaged endocytosed Syp-SEP after the rapid extracellular pH change to 6.0 at a higher magnification using 150× objective lens to examine where endocytosed Syp-SEP was localized in relation to AZLM (Figure 3A). Line scans across CAST-RFP and Syp-SEP signals from a representative case showed that the position of endocytosed Syp-SEP signal peaks at 3.24 and 7.24 s after the stimulation were about 170 nm from the CAST-RFP signal peak (Figures 3A,B). However, Syp-SEP signals at pH 6.0 were scattered, and there were multiple intensity peaks in several trials. In such cases, we attempted to divide the Syp-SEP areas by watershed lines (Figure 3C). The centroid of the Syp-SEP signal after the stimulation at pH7.4, which reflects the exocytosis of a large number of synaptic vesicles, was about 86 ± 13 nm (*n* = 45 clusters) inside of the edge of the CAST-RFP signal, whereas that at pH 6.0, which reflects endocytosis, was about 100 ± 28 nm (*n* = 69 clusters) and 73 ± 27 nm (*n* = 62 clusters) outside of the edge of the CAST-RFP signal at 3.24 s and 7.24 s after the stimulation (Figure 3D). The latter two values were significantly different from the former (*p* < 0.001, Steel-Dwass’s test). Thus, Syp-SEP signals at pH 6.0, which reflect endocytosis-related vesicles, were found in the periphery of AZLM. The centroid of the Syp-SEP signal at pH 6.0 does not always correspond to the center of an endocytosed vesicle, because the Syp-SEP signal might have come from multiple vesicles that were not separated by the watershed lines. On the other hand, we previously reported that Syp-SEP is released inside AZLM by an electric field stimulation (Funahashi et al., 2018), which is consistent with the present results. Additionally, the CAST-RFP signal was stable and moved little during the recording: the distance between the centroid of the CAST-RFP signal before and after the stimulation was 30.8 ± 4.2 nm (Figure 3E).

**Figure 4:**
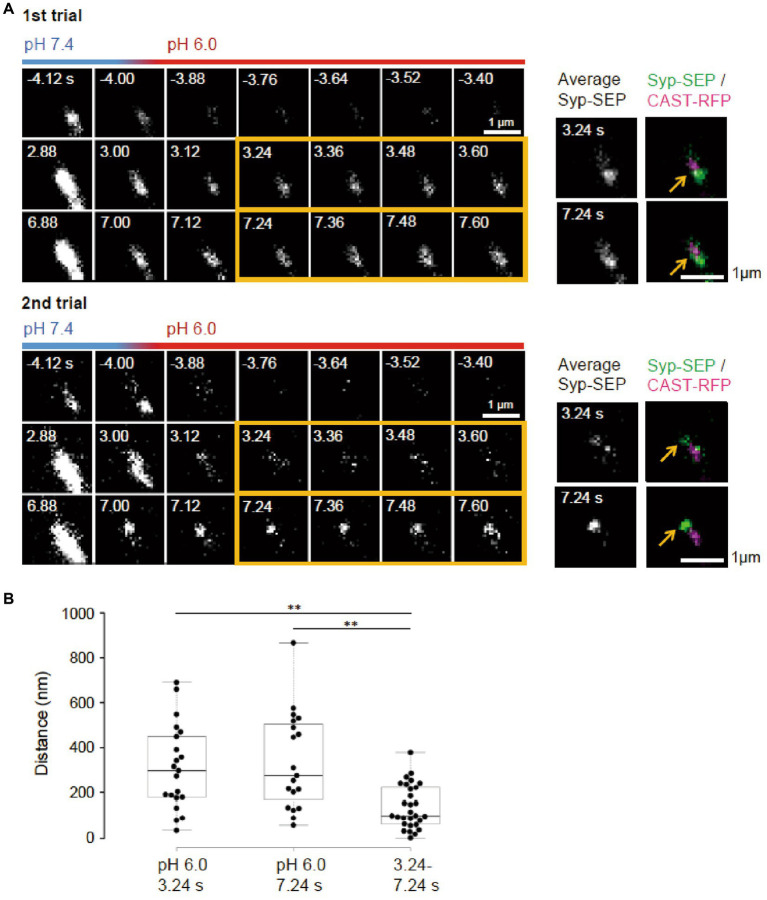
Locations of endocytosed Syp-SEP signals in different trials. **(A)** Representative high magnification images of Syp-SEP at pH 6.0 after the 50 pulses stimulation in different trials using the same sample. The right images are averages of the images enclosed by the yellow lines and were merged with an image of CAST-RFP signals. Arrows indicate positions of Syp-SEP around an AZLM. The signal locations were different between 1st and 2nd trials. **(B)** Displacement of centroids of Syp-SEP signals at pH 6.0 in different trials (3.24 s, 7.24 s) or over a time range (3.24–7.24 s) in a trial (3.24 s, *n* = 21 clusters, 7.24 s, 19 clusters, 3.24–7.24 s, 29 clusters, 11 cells). ** indicate *p* < 0.01.

It remains unclear whether synaptic vesicle proteins were endocytosed in specialized regions. To examine if there were such endocytic zones around an active zone, the same neuron was repeatedly stimulated (Figure 4A). Syp-SEP signals at pH 6.0 were found at different locations in different trials. The distance between the centroid of the Syp-SEP signals in the first trial and the nearest centroid in the second trial was 310 ± 42 nm (*n* = 21 clusters) at 3.24 s and 340 ± 50 nm (*n* = 19 clusters) at 7.24 s (Figure 4B), although all of the centroids were distributed in the periphery of AZLM. The average distance between the centroids at 3.24 s and 7.24 s in the same trial was 140 ± 18 nm (*n* = 29 clusters), which was smaller than the distance for different trials (*p* < 0.01, Steel-Dwass’s test). These results suggest that endocytosis locations change in different trials and that a part of the Syp-SEP signal recorded at 7.24 s might have been derived from Syp-SEP endocytosed before 3.24 s.

### Clathrin-dependent and -independent endocytosis

**Figure 5:**
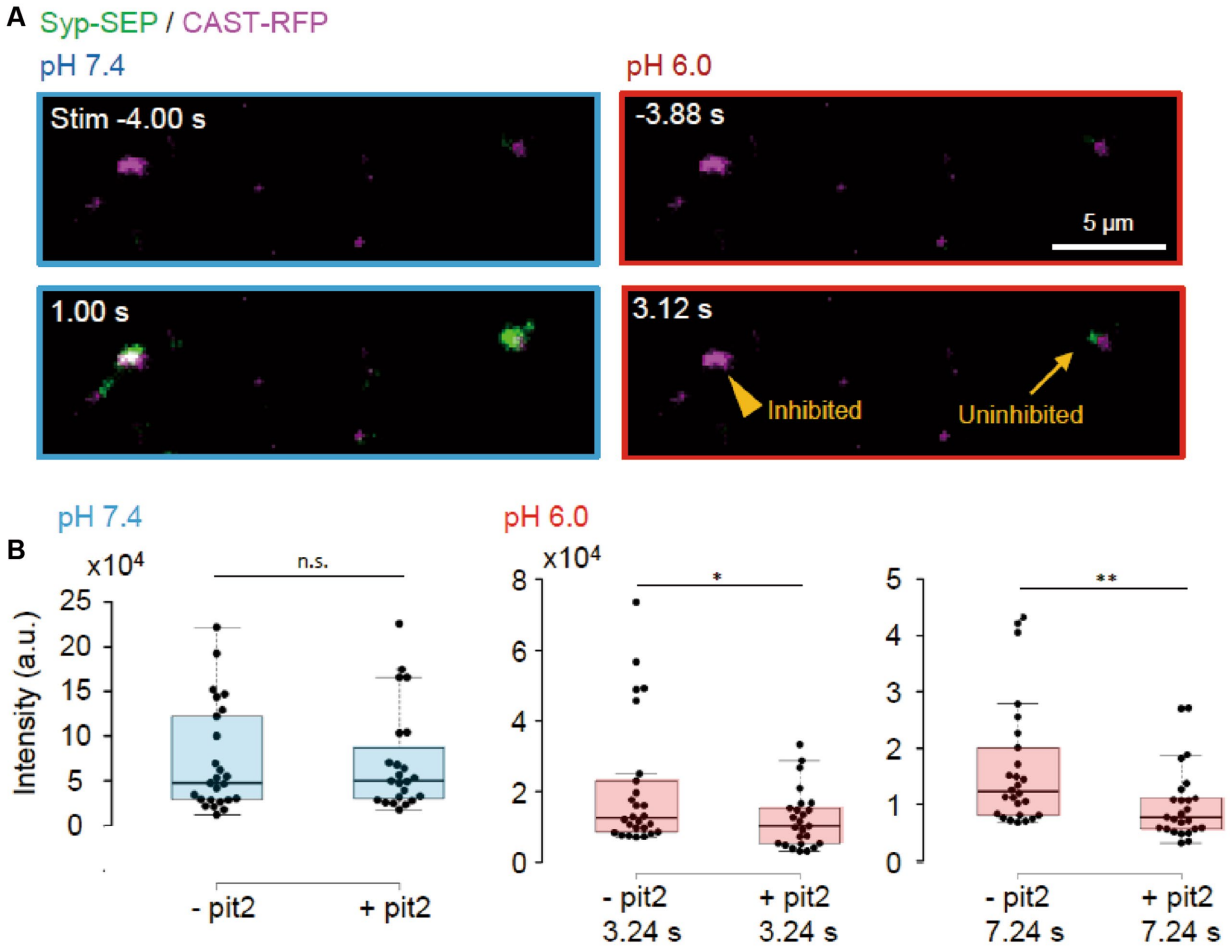
Clathrin-dependent and -independent endocytosis. **(A)** Representative images of Syp-SEP and CAST-RFP signals at pH 7.4 or 6.0 before and after the 50 pulses stimulation in the presence of pit2. While endocytosis was inhibited in some AZLM (arrowhead, pH 6.0, 3.12 s) after the exocytosis of Syp-SEP (pH 7.4, 1.0 s), endocytosed Syp-SEP was detected in other AZLM (arrow, pH 6.0, 3.12 s). **(B)** Quantification of Syp-SEP at pH 7.4 at 1 s after the stimulation (blue) and at pH 6.0 at 3.24 or 7.24 s after the stimulation (red) without or with pit2 (−pit2, *n* = 26 clusters; +pit2, 25 clusters, 7 cells). * and ** indicates *p* < 0.05 and 0.01 respectively, n.s., not significant.

**Figure 6:**
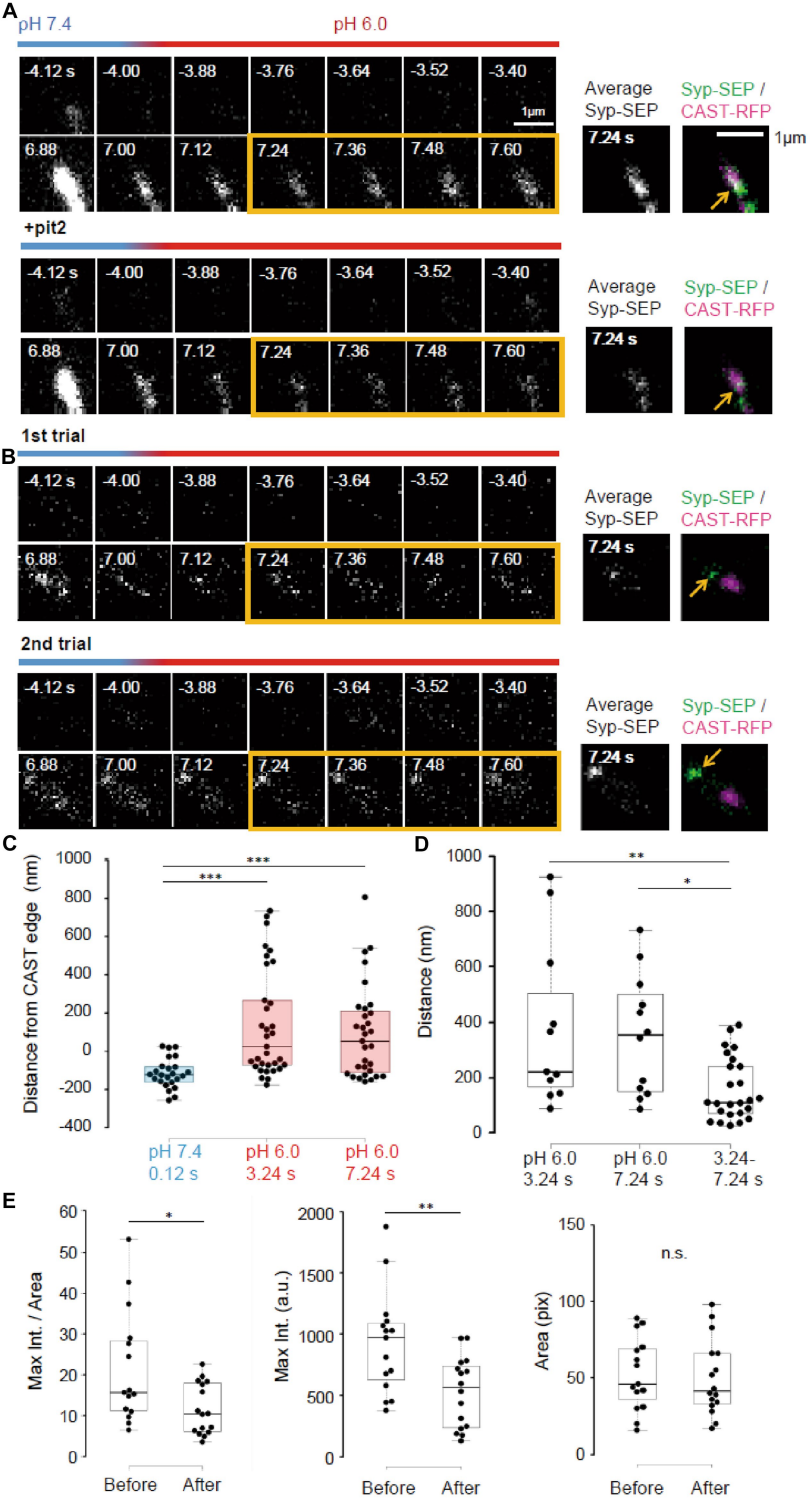
Pit2-resistant endocytosis. **(A)** Representative high magnification images of Syp-SEP signals at pH 7.4 and 6.0 before and after the 50 pulses stimulation without pit2 (upper images) and after application of pit2 (lower images) in the same sample. The right images are averages of the images enclosed by the yellow lines and were merged with an image of CAST-RFP signals. The Syp-SEP signal indicated by an arrow in the top image was clearly diminished after addition of pit2 (bottom image). **(B)** Representative images of Syp-SEP signals at pH 7.4 and 6.0 before and after the stimulation in the presence of pit2 in different trials using the same sample. The right images are averages of the images enclosed by the yellow lines and were merged with an image of CAST-RFP signals. Arrows indicated Syp-SEP in different locations in different trials. **(C)** Location of centroids of Syp-SEP signals at pH 7.4 at 0.12 s after the stimulation and at pH 6.0 at 3.24 s or 7.24 s after the stimulation in relation to the edge of the CAST-RFP signal (0.12 s, *n* = 24 clusters, 3.24 s, 33 clusters, 7.24 s, 31 clusters, 8 cells). **(D)** Displacement of centroids of Syp-SEP signal at pH 6.0 in different trials (3.24 s, 7.24 s) or over a time range (3.24–7.24 s) in a trial (3.24 s, *n* = 11 clusters, 7.24 s, 12 clusters, 3.24–7.24 s, 25 clusters, 8 cells). **(E)** Quantification of the maximum intensity, the area and the maximum intensity divided by the area of Syp-SEP signals at pH 6.0 at 7.24 s after the stimulation (Before *n* = 15 clusters; After, 16 clusters, 6 cells). *, **, and *** indicate *p* < 0.05, 0.01 and 0.001, respectively. n.s., not significant.

Next, we tried to determine whether the endocytosis we studied was clathrin-dependent or -independent using the clathrin inhibitor pitstop2 (pit2) at RT (Figures 5A, 6A). When pit2 was added to the extracellular solution, the Syp-SEP signal intensity at pH 6.0 after the stimulation became smaller: at 3.24 s, the intensity was 20,700 ± 3,600 (*n* = 26 clusters) without pit2 and 12,200 ± 1700 with pit2 (*n* = 25 clusters, *p* < 0.05, Mann–Whitney U test); at 7.24 s, it was 16,300 ± 2,200 without pit2 (*n* = 26 clusters) and 10,100 ± 1,300 with pit2 (*n* = 26 clusters, *p* < 0.01, Mann–Whitney U test). The Syp-SEP signal intensity at pH 7.4 did not change significantly by pit2 application (Figure 5B, *p* = 0.67, Mann–Whitney U test). The pit2-sensitive component of the Syp-SEP signal at pH 6.0 presumably related to CME, whereas endocytosis that was not inhibited by pit2 was clathrin-independent. Previous studies reported that a high-frequency stimulation triggers ADBE (Cheung and Cousin, 2013; Kokotos et al., 2018; Renard and Boucrot, 2021). It has been proposed that ADBE is triggered by a high load of membrane addition into the presynaptic plasma membrane through the exocytosis of a large number of synaptic vesicles and that the elevated concentration of cytoplasmic calcium in presynaptic terminals plays a role in this process. A field stimulation of 50 pulses at 50 Hz released most vesicles in a readily releasable pool. Therefore, the clathrin-independent endocytosis we recorded here is likely to correspond to ADBE.

Next, we took higher magnification images of Syp-SEP (Figures 6A,B). The centroids of the Syp-SEP signal at pH 6.0 detected in the presence of pit2, which presumably reflected ADBE, were located 139 ± 49 nm (*n* = 33 clusters) outside of the edge of the CAST-RFP signal at 3.24 s and 97 ± 44 nm (*n* = 31 clusters) at 7.24 s, but at pH 7.4 and 1 s after the stimulation, the centroid was 115 ± 16 nm (*n* = 24 clusters) inside of the edge (Figure 6C, *p* < 0.001 for both, Steel-Dwass’s test). Thus, ADBE are also likely to occur at the periphery of the CAST-RFP signal.

To examine whether the region where ADBE occur is fixed, the stimulation was applied repeatedly (Figure 6B). The distance between the centroid of the Syp-SEP signal in the first trial and the nearest centroid in the second trial was 378 ± 94 nm (*n* = 11 clusters) at 3.24 s and 351 ± 65 nm (*n* = 12 clusters) at 7.24 s (Figure 6D), which are significantly smaller than the distances recorded at 3.24 s and 7.24 s in a single trial (158 ± 23 nm, *n* = 25 clusters, *p* < 0.01 and 0.05, Steel-Dwass’s test). These results suggest that there are multiple ADBE zones around an active zone and which zone is used for ADBE varies trial to trial.

To address whether the ADBE-dependent and CME-dependent intracellular Syp-SEP spatial distribution patterns were different, we compared the Syp-SEP signal distribution characteristics before and after pit2 application. We found that the maximum Syp-SEP signal intensity and that divided by the Syp-SEP signal-positive area at 7.24 s after the stimulation were significantly larger than those before pit2 application (Figure 6E, *n* = 15 clusters before application and *n* = 16 clusters after, signal intensity, *p* < 0.05 Mann–Whitney U test; signal intensity / area, *p* < 0.01 Student’s paired t test). The Syp-SEP signal-positive areas were not significantly different with or without pit2. These results suggest that the intracellular Syp-SEP cluster formed through CME might be more concentrated than the cluster formed through ADBE. However, it might be also possible that some CME-mediated and ADBE-mediated Syp-SEP signals were not spatially separated and that in such cases the recorded maximum Syp-SEP signal intensity without pit2 was the sum of the CME- and ADBE-mediated signals.

### Temperature-sensitive endocytosis occurs immediately after the stimulation

**Figure 7:**
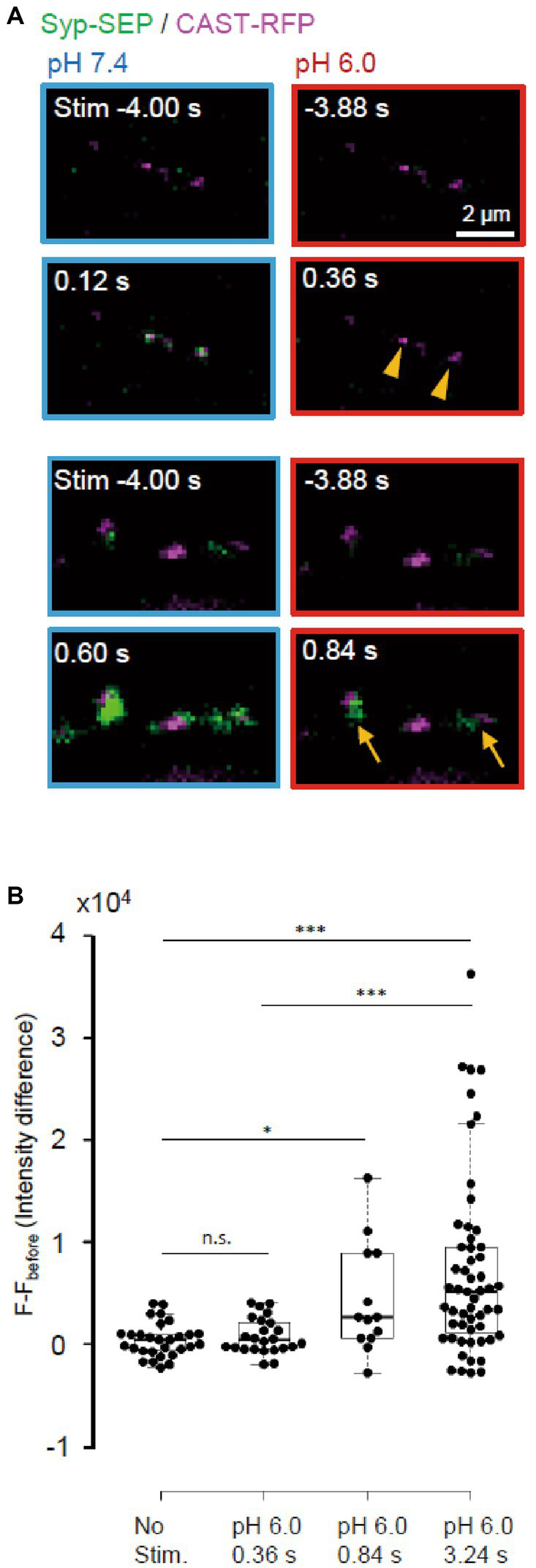
Absence of ultrafast endocytosis at RT. **(A)** Representative images of Syp-SEP at pH 7.4 at 4 s before and at 0.12 s (top) or 0.60 s (bottom) after the 5 pulses stimulation, and at pH 6.0 at 3.88 s before and at 0.36 s (top) or 0.84 s (bottom) after the stimulation. All Syp-SEP signals were merged with CAST-RFP signals. Endocytosed Syp-SEP signals were detected not at 0.36 s (arrowheads) but at 0.84 s (arrows) after the stimulation. **(B)** Quantification of Syp-SEP at pH 6.0 without stimulation (*n* = 29 clusters, 10 cells), and at 0.36 s (25 clusters, 7 cells), 0.84 s (13 clusters, 6 cells) and 3.24 s (55 clusters, 20 cells) after the 5 pulses stimulation. The intensity value (F – F_before_) was calculated by subtracting the value 3.88 s before the stimulation (in no stimulation, the value 4.24 s before the timing of stimulation onset was subtracted). Thus, when the intensity value before the stimulation was comparably large due to the signal noise and that after was comparably small, the subtracted and presented value took small negative value. * and *** indicates *p* < 0.05 and 0.001 respectively; n.s., not significant.

Next, we examined whether endocytosis could be detected immediately after the stimulation (Figure 7). UFE, which is faster than other types of endocytosis, has been reported to occur around active zones at 34°C (Watanabe et al., 2013). After a single stimulus, the synaptic vesicle membrane is recovered at sites in the vicinity of the active zone within 100 ms independently of clathrin. It is not known whether UFE mediates the recovery of synaptic vesicle proteins, which should be an important function of endocytosis at presynaptic terminals (Watanabe and Boucrot, 2017). We attempted to determine whether UFE retrieves laterally diffused Syp-SEP after exocytosis (Gimber et al., 2015; Funahashi et al., 2018) in a living neuron. At RT, we detected the Syp-SEP signal increases at pH 6.0 at 0.84 s after the stimulus but not at 0.36 s after (Figure 7B, *p* < 0.001 and 0.05, Steel-Dwass’s test). Thus, very fast endocytosis was not detected at RT, suggesting that UFE was unlikely to occur at RT, which is consistent with previous reports (Watanabe et al., 2013).

**Figure 8:**
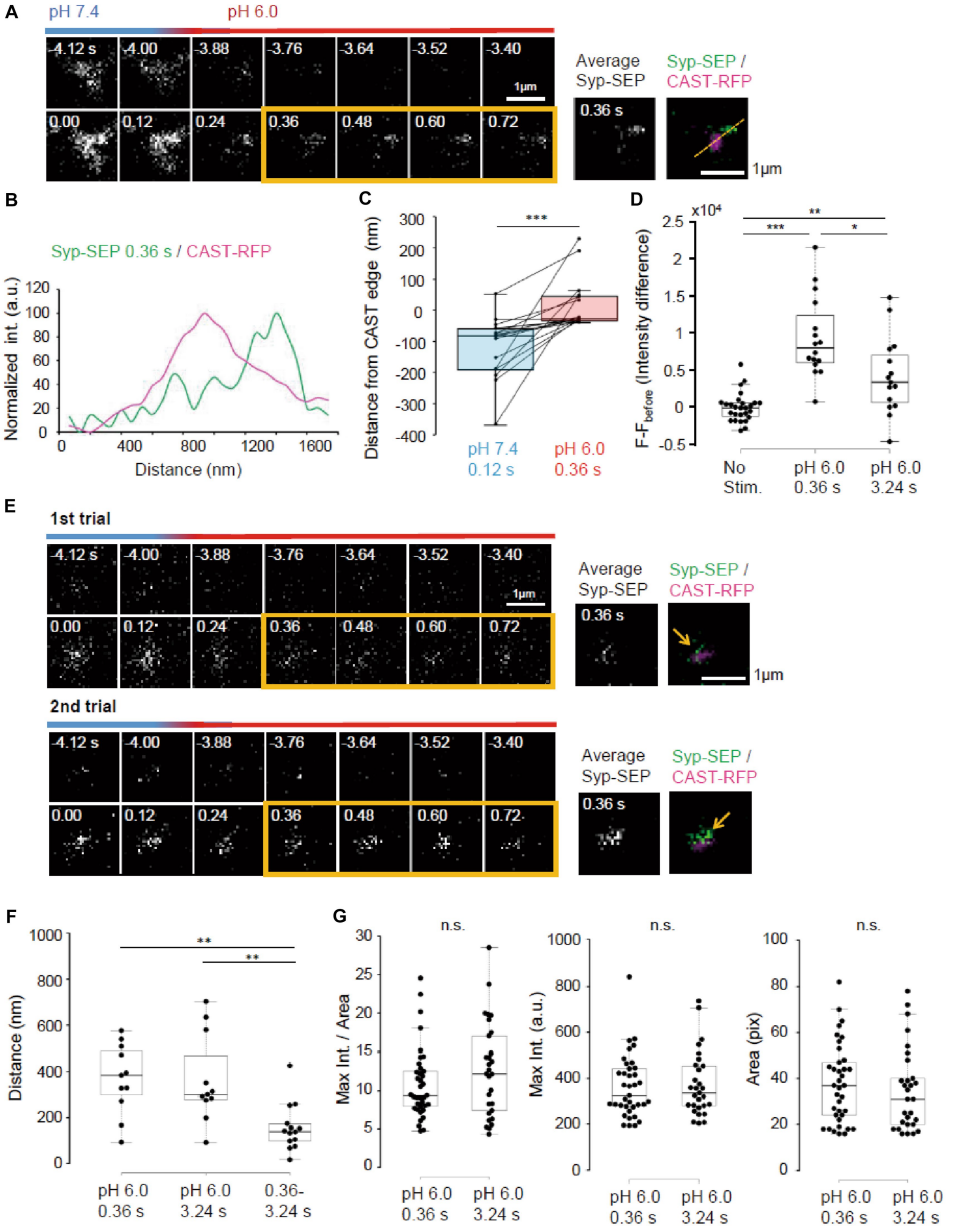
Ultrafast endocytosis at PT. **(A)** Representative high magnification images of Syp-SEP at pH 7.4 and 6.0 before and immediately after the 5 pulses stimulation at PT. The right images are averages of the images enclosed by the yellow lines and were merged with an image of CAST-RFP signals. **(B)** Line-scan of Syp-SEP and CAST-RFP signal intensities on the yellow broken line shown in **(A)**. **(C)** Location of centroids of Syp-SEP signals at pH 7.4 at 0.12 s after the stimulation and at pH 6.0 immediately after the stimulation (the centroid calculated from an averaged image between 0.36 s and 0.72 s) in relation to the edge of CAST-RFP signal (0.12 s, *n* = 17 clusters, 0.36 s, 17 clusters, 7 cells). The data obtained in a trial were connected by a line. **(D)** Quantification of Syp-SEP signal intensities at pH 6.0 without stimulation and at 0.36 s or 3.24 s after the stimulation (No stim., *n* = 7 clusters; 0.36 s, 16 clusters; 3.24 s, 15 clusters, 8 cells). The intensity value (F – F_before_) was calculated by subtracting the value 3.76 s before the stimulation (in no stimulation, the value 3.76 s before the timing of stimulation onset was subtracted). Thus, when the intensity value before the stimulation was comparably large due to the signal noise and that after was comparably small, the subtracted and presented value took small negative value. **(E)** Representative images of Syp-SEP at pH 7.4 and 6.0 before and immediately after the stimulation in different trials using the same sample. The right images are averages of the images enclosed by the yellow lines and were merged with an image of CAST-RFP. Arrows indicate Syp-SEP signal. The signal locations were different between 1st and 2nd trials. **(F)** Displacement of centroids of Syp-SEP signals at pH 6.0 in different trials (0.36 s, 3.24 s) or over a time range (0.36–3.24 s) in a trial (0.36 s, *n* = 11 clusters, 3.24 s, 11 clusters, 0.36–3.24 s, 14 clusters, 4 cells). **(G)** Quantification of the maximum intensity, the area and the maximum intensity divided by the area of Syp-SEP signals at pH 6.0 at 0.36 s and 3.24 s after the stimulation (0.36 s, *n* = 37 clusters, 3.24 s, 29 clusters, 9 cells). *, **, and *** indicate *p* < 0.05, 0.01 and 0.001, respectively. n.s., not significant.

To make UFE more likely to occur, the temperature was raised to near physiological temperature (PT, 31–32°C). We also reduced the number of stimulation pulses to 5. At PT, the Syp-SEP signal was recorded when pH was changed at 120 ms after the onset of the stimulation (Figure 8A). Line scans across CAST-RFP and Syp-SEP images at high magnification show the position of the endocytosed Syp-SEP signal peak at 0.36 s after the stimulation was about 500 nm from the position of the CAST-RFP signal peak (Figure 8B). The Syp-SEP signal centroid at pH 6.0 was 20 ± 21 nm (*n* = 17 clusters) outside the edge of the CAST-RFP signal at 0.36 s, but 110 ± 25 nm (*n* = 17 clusters) inside the CAST-RFP signal edge at pH 7.4 at 0.12 s after the stimulation (Figure 8C, *p* < 0.001, Wilcoxon signed-rank test). Importantly, the Syp-SEP signal intensity at pH 6.0 was larger at 0.36 s after the stimulation than that at 3.24 s (Figure 8D, *p* < 0.05, 0.01, and 0.001, Steel-Dwass’s test). These results suggest that temperature-dependent UFE occurs immediately after the stimulation, and it dominates when the number of stimulation pulses is small at PT. We did not observe a clear Syp-SEP signal at pH 6.0 inside of AZLM at 0.36 s after the onset of stimulation.

Next, we addressed whether the region where UFE occurred was fixed by applying the stimulation repeatedly (Figure 8E). The distance between the centroid of the Syp-SEP signal in the first trial and the nearest centroid in the second trial at 0.36 s was 369 ± 46 nm (*n* = 11 clusters), which was significantly larger than the distance between the centroids of the Syp-SEP signal at 0.36 s and 3.24 s in a single trial (Figure 8F, 157 ± 27 nm, *n* = 14 clusters, *p* < 0.01, Steel-Dwass’s test). These results suggest that there are multiple UFE zones around an active zone and that the Syp-SEP signal recorded at 3.24 s included Syp-SEP endocytosed before 0.36 s.

Lastly, the intracellular Syp-SEP signal spatial distribution after UFE was analyzed. The maximum Syp-SEP signal intensity divided by the Syp-SEP signal-positive area at 0.36 s was 8.3 ± 0.6 and at 3.24 s it was 9.6 ± 0.9 (Figure 8G, *p* = 0.30, Mann–Whitney U test). Considering that Syp-SEP is about 32% brighter at RT than at PT, the UFE-dependent maximum Syp-SEP signal intensity divided by the signal-positive area at RT at 0.36 s was 10.9 ± 0.8, which was not significantly different from the value at 7.24 s after 50 pulses stimulation at RT with pit2 (11.5 ± 1.6, *p* = 0.98, Steel-Dwass’s test), but smaller than that without pit2 (21.6 ± 3.6, *p* < 0.01, Steel-Dwass’s test). We could not separate the Syp-SEP signal-positive area at 0.36 s using watershed lines.

## Discussion

### Methods to study endocytosis in presynaptic terminals

We have established a new experimental method for the live-cell imaging of endocytosed synaptic vesicle membrane proteins at multiple time points after the exocytotic fusion of vesicles triggered by electrical stimulation. By combining the rapid extracellular pH exchange method and TIRFM observation around AZLM formed on a glass surface, we recorded clathrin-dependent and -independent endocytosis-related signals at RT and also temperature-sensitive ultrafast endocytosis-related signals. This method enabled us to directly observe the spatial pattern of the endocytosed Syp-SEP signal around AZLM repeatedly, which is in principle inaccessible with EM and electrophysiological capacitance measurements. Morphological observations by EM need fixation of the preparation and thus only provide data at one time point for a preparation (Watanabe et al., 2013). Electrophysiological recordings provide continuous data from a live preparation with a high temporal resolution, but they cannot provide information about the localization of synaptic vesicle proteins or the morphological properties of endocytic events (Delvendahl et al., 2016).

### Fluorescence imaging studies

Various types of fluorescence imaging techniques have been used to study exocytotic and/or endocytic processes in presynaptic terminals (Welzel et al., 2013; Egashira et al., 2015; Midorikawa and Sakaba, 2015; Okamoto et al., 2016; Guillaud et al., 2017; Soykan et al., 2017; Mori et al., 2021). Our method is an advancement of these studies in the following two aspects. First, the signal-to-noise ratio and z-axis spatial resolution were improved by applying TIRFM to AZLM. As described, using TIRFM, we could record signals from single SEP molecules (Figure 1E). In addition, a near two-dimensional analysis of the distribution of endocytosed synaptic proteins was performed by forming AZLM parallelly on the glass surface. Second, extracellular pH exchange using U-tube enabled fast extracellular pH switching and the recording of endocytosed synaptic vesicle proteins at different times repetitively.

We used a 150× optical lens with a high numerical aperture (1.45) to obtain a high XY spatial resolution; nevertheless, the resolution was restricted by the diffraction limit. Super resolution techniques, such as stochastic optical reconstruction microscopy (STORM), photoactivated localization microscopy (PALM), stimulated emission depletion microscopy (STED) and/or structured illumination microscopy (SIM), have been used to study exo- and endocytic processes in presynaptic terminals and endocrine cells at higher spatial resolution (Schermelleh et al., 2010; Maglione and Sigrist, 2013; Sigal et al., 2018; Schermelleh et al., 2019). Each of these methods has merits and demerits. Future studies combining our method and super-resolution imaging should provide higher quality data and more detailed information about the recycling processes of synaptic vesicles.

### Identity of Syp-SEP signal recorded at pH 6.0

The Syp-SEP signal we recorded at pH 6.0 should have come from intracellular vesicles with an intraluminal neutral pH located within about 200 nm from the glass surface, which corresponds to the TIRFM visualization zone. Considering the thickness of the NLG coating and the extracellular space and thickness of plasma membrane, we estimate that the presynaptic intracellular space was about >50 nm above the glass surface. Most of the Syp-SEP signal observed at pH 6.0 likely came from one or multiple vesicles endocytosed just before the pH exchange, before intraluminal acidification and before the vesicles moved out of the TIRFM visualization zone. The Syp-SEP signal could have also come from endosomes having near neutral intraluminal pH and/or endoplasmic reticulum. Therefore, we subtracted the Syp-SEP image recorded before the stimulation from that recorded after the stimulation to exclude intracellular Syp-SEP signals insensitive to the stimulation. The Syp-SEP signal may also have come from vesicles endocytosed sometime before but moved into the visualization zone from the outside. In order not to include such signals in the analysis, we excluded signals which appeared after the pH change to 6.0. It has been reported that the acidification of endocytosed vesicles takes 3–15 s (Atluri and Ryan, 2006; Granseth et al., 2006; Egashira et al., 2015). It has also been shown that synaptic vesicles can enter and exit the TIRFM visualization zone in about 100 ms (Midorikawa and Sakaba, 2015). Our observations indicated that the Syp-SEP signal recorded at pH 6.0 at 7.24 s includes partial signals from Syp-SEP endocytosed before 3.24 s.

### CME and ADBE

Previous studies have demonstrated the presence of CME and clathrin-independent endocytosis at mammalian central synapses. CME was originally thought to be the predominant endocytic mechanism for recycling synaptic vesicles because of the sensitivity to perturbation of clathrin or clathrin-associated proteins, and its time constant was reported to be 15–30 s at RT (Granseth et al., 2006). Here, we showed that the exocytosed Syp-SEP signal after 50 pulses at 50 Hz decreased with a similar time constant of about 20 s (Figure 2A). The speed of CME is thought to be limited by the selection and gathering processes of clathrin and its associated proteins including cargo receptors (Ehrlich et al., 2004; Taylor et al., 2011; Watanabe and Boucrot, 2017). Therefore, not all endocytosis detected at 3.24 and 7.24 s after the 50 pulses stimulation was unlikely to be CME (Figures 3A, 4A).

When a strong stimulation such as 50 Hz or high K^+^ solution is applied to neurons, a large area of the plasma membrane is internalized through the clathrin-independent mechanism ADBE (Clayton and Cousin, 2009). Synaptic vesicles are then regenerated from endosomes through a clathrin-dependent process (Kononenko et al., 2014; Watanabe et al., 2014). Recent studies have suggested that ADBE is the dominant endocytosis mechanism during and after high frequency presynaptic activation at PT. The major role of ADBE is not the immediate restoration of synaptic vesicles but it may involve the clearance of fusion sites for later exocytosis (Watanabe et al., 2014; Soykan et al., 2017; Chanaday et al., 2019; Ivanova and Cousin, 2022). On the other hand, CME might contribute to the reformation of synaptic vesicles more directly by gathering membrane proteins of synaptic vesicles through clathrin-associated adaptor proteins over a slower time course. Synaptophysin is diffused out of AZLM following exocytosis (Gimber et al., 2015; Funahashi et al., 2018). We showed that the maximum Syp-SEP signal intensity at pH 6.0 divided by the Syp-SEP-positive area was significantly larger before pit2 application than after, which suggests that CME-dependent intracellular vesicles concentrate Syp more than ADBE-dependent ones. This different effect by the two types of vesicles might be caused by trapping Syp through adaptor proteins in CME but not in ADBE. Thus, the density of Syp-SEP in CME-derived vesicles might be higher than that in ADBE-derived vesicles. In addition, a part of ADBE-derived large vesicle could be far from the bottom glass surface and out of TIRFM visualization zone, which might have made the Syp-SEP signal intensity weaker. It is to be noted that there is a possibility that in some cases CME-mediated and ADBE-mediated Syp-SEP signals were closely localized, such that both contributed to the signal intensity of a particular pixel.

### UFE

An innovative flash-and-freeze approach using optogenetics and freezing neurons at defined time points after the stimulation revealed the existence of UFE. UFE occurs at sites lateral to fusion sites (typically <200 nm) within 100 ms after a stimulation, does not require clathrin, and is temperature dependent (Watanabe et al., 2013, 2014). The amount of membrane internalized by UFE equals the membrane exocytosed (Watanabe et al., 2013). It was suggested that any excess membrane must be removed rapidly from the plasma membrane to restore fusion sites and keep the membrane area and tension constant during a high load of membrane turnover (Rosenmund et al., 1993), something UFE seems to manage (Watanabe and Boucrot, 2017). However, it was unknown whether UFE contributes to the recovery of vesicle proteins. We found that endocytic vesicles contained Syp-SEP at 0.36 s after the onset of a stimulation at PT (Figure 8A). The maximum Syp-SEP signal intensity divided by the signal-positive area of UFE was comparable to that of ADBE after compensating for temperature effects on SEP fluorescence, suggesting that Syp-SEP densities were similar in UFE-mediated and ADBE-mediated intracellular vesicles (Figures 6E, 8G).

We would like to note that Syp-SEP signal recorded at 0.36 s might have come from single vesicle in some cases because of the following reason. UFE forms a vesicle of about 100 nm diameter (Watanabe et al., 2013) and the diameter of synaptic vesicle is about 50 nm. Thus, UFE internalizes membrane area corresponding to 4 synaptic vesicles. On the other hand, according to our previous study on synaptic vesicle exocytosis in AZLM, 5 pulses stimulation at 50 Hz seems to induce fusion of around 6 synaptic vesicles (Funahashi et al., 2018).

### K & R

An alternative fast endocytosis pathway, K&R, was reported at neuronal synapses and in neuroendocrine cells (Gandhi and Stevens, 2003; Zhang et al., 2009), although whether K&R occurs in the mammalian central nervous system is controversial (He and Wu, 2007). The transient opening and closing of fusion pores during intense stimulation by high K^+^ solution was reported using quantum dots (Zhang et al., 2009). However, the large size of quantum dots might have affected fusion pore openings and vesicle collapse (Dittman and Ryan, 2009). K&R should take place at exocytosis sites within an active zone immediately after the exocytosis. However, we did not record events that unequivocally reflected K&R in AZLM, suggesting K&R was rare in our preparations.

### Endocytosis sites

One merit of our experimental system is that AZLM can be repeatedly stimulated and endocytosed SEP-tagged synaptic proteins can be imaged after each stimulation. The results show that under different conditions (5 or 50 pulses at RT or PT), the majority of endocytosed Syp-SEP signals was located <200 nm away from the edge of AZLM, but the positions varied trial to trial. Proteins involved in endocytosis might not be immobilized. However, the existence of hotspots, where a particular type of endocytosis such as UFE, ADBE and/or CME preferentially occurs, cannot be excluded, since the number of trials in our experimental protocol was limited due to the bleaching of Syp-SEP and CAST-RFP. As for the exocytosis of synaptic vesicles, the existence of several hot spots within an active zone has been suggested (Maschi and Klyachko, 2017; Funahashi et al., 2018).

### Caveat, remaining questions and future advancement

One caveat of this study is that AZLM is an artificial structure and may have characteristics that are different from normal presynaptic active zones. However, our previous study demonstrated the accumulation of various active zone proteins in AZLM and that the electrical stimulation triggered fusion of synaptic vesicles in AZLM, suggesting that AZLM shows essential properties of active zones and is a reliable model (Funahashi et al., 2018). Future study comparing the results obtained in AZLM with those in normal synapses in detail might provide useful information. It might be also possible to induce AZLM with somewhat different characteristics such as that in large or inhibitory presynaptic terminals using other synaptic adhesion molecules (Südhof, 2021). Comparison of properties among different types of AZLM could be interesting.

Another limitation of the present method is XY spatial resolution is restricted by the diffraction limit and is inferior to super resolution techniques such as STED, STORM, PALM and SIM (Schermelleh et al., 2010, 2019; Maglione and Sigrist, 2013; Sigal et al., 2018). Future studies combining the present method and one or some of super-resolution imaging techniques would provide more detailed information about the recycling processes of synaptic vesicle proteins. Here, we have examined only synaptophysin as a synaptic vesicle protein. Other synaptic vesicle proteins such as synaptotagmin, synaptobrevin or vglut1 might show different intracellular distribution after endocytosis, because each might be trapped on the membrane of endocytosed vesicles through different adaptor proteins (Cousin, 2017). Thus, extending the analyses to other types of synaptic vesicle protein would contribute to better understanding of retrieval processes of synaptic vesicles.

## Data availability statement

The original contributions presented in the study are included in the article/supplementary material, further inquiries can be directed to the corresponding author.

## Ethics statement

The animal study was approved by Local committee for handling experimental animals in the Graduate School of Science, Kyoto University. The study was conducted in accordance with the local legislation and institutional requirements.

## Author contributions

HT: Conceptualization, Formal analysis, Funding acquisition, Investigation, Methodology, Software, Validation, Writing – original draft, Writing – review & editing. JF: Methodology, Writing – review & editing. TH: Conceptualization, Funding acquisition, Supervision, Writing – review & editing.
